# Intelligent Foreign Particle Inspection Machine for Injection Liquid Examination Based on Modified Pulse-Coupled Neural Networks

**DOI:** 10.3390/s90503386

**Published:** 2009-05-07

**Authors:** Ji Ge, YaoNan Wang, BoWen Zhou, Hui Zhang

**Affiliations:** College of Electrical and Information Engineering, Hunan University, China; E-Mail: yaonan@hnu.cn (Y-N.W.)

**Keywords:** Intelligent inspection machine, foreign particle detection, modified PCNN, injection quality inspection, image processing, illumination styles

## Abstract

A biologically inspired spiking neural network model, called pulse-coupled neural networks (PCNN), has been applied in an automatic inspection machine to detect visible foreign particles intermingled in glucose or sodium chloride injection liquids. Proper mechanisms and improved spin/stop techniques are proposed to avoid the appearance of air bubbles, which increases the algorithms' complexity. Modified PCNN is adopted to segment the difference images, judging the existence of foreign particles according to the continuity and smoothness properties of their moving traces. Preliminarily experimental results indicate that the inspection machine can detect the visible foreign particles effectively and the detection speed, accuracy and correct detection rate also satisfying the needs of medicine preparation.

## 1. Introduction

Injection liquid clarity is one of the vital indexes in clinical treatment using fluids. The presence of visible foreign particles, which can not be metabolized by human beings in injection liquids is prohibited [1]. However, different kinds of foreign substances such as rubber chips, chemical fibers, glass fragments, calcium carbonate and other crystalline particles [2] appear due to problems with injection bottle quality, packing procedures, collisions, filtration or filling. They can cause thrombus, phlebitis, tumor, anaphylactic reaction or even death when these kinds of particles are injected into the vein. Thus, foreign particles in injection liquids are now a matter of social concern which is often reported in the mass media. Thus, it was reported recently that more than 75% medicine recalls were related to the presence of foreign substances. Pharmaceutical corporations have been taking active measures against this problem to avoid damage to their public image and economic losses caused by recalls. Nevertheless, according to the statistics [3] taken by China Pharmaceutical Industry Association (CPIA), 99.6% pharmaceutical corporations in China use a light inspection method carried out by workers [1]: inspectors put the injection bottle under a special lamp housing, rotate and turn it over gently, suspending any visible foreign substance present in the transfusion and then deciding whether it is acceptable or not based on their inspection experience. This method is simple, but the inspection efficiency and repeatability are poor, with omission rates increasing synchronously due to workers' tiredness.

A visible foreign particles inspection system was previously developed and has been utilized in an actual product line [4,5]. In this system, an injection container such as an ampoule is rotated at high speed and stopped abruptly. The ampoule forms a vortex due to inertia. The moving foreign substances can be distinguished from stationary scratches on the surface of the ampoule by applying image processing techniques. Detection in rectangular plastic bottles of medicinal solutions based on real-time image processing are proposed in [6]. Some experiments have been performed on discrimination of particle in size and shape [7] and visualization of the spatial behavior of particles [8]. Developmental research for detecting impurity particles in a water supply has been described in [9].

Currently we are developing an Automatic Inspection Machine (AIM) equipped with CCD cameras. Since an ampoule or a vial is small and simple in shape, having a smooth surface, it is easy to gather any foreign substances present in the center by forming a vortex in the solution and thus distinguish them from scratches and other defects of the surface of the container. Nevertheless, in our machine, the detection target is mass transfusion and it is commonly contained in a glass or plastic bottle. Sometimes the cross sections of the bottle are circular, oval, rectangular or hexagonal and they can have a complicated surface such as embossed symbols and graduations on the surface, which makes it difficult to create a vortex and distinguish the foreign particles from uneven surface features.

Hence, image segmentation and recognition of foreign particles is the key module of AIM. A large number of different image segmentation techniques are currently available. Basic algorithms like thresholding, edge detection and region growing are described in [10]. Although simple to implement, these methods usually have serious shortcomings when employed in practical situations. The most obvious case is thresholding, which can only be used to segment an image if the segments consist of non-overlapping pixel intensity ranges, which is rarely the case. Edge detection and region growing can tolerate some intensity overlap between the different segments, but can fail if the edges between the segments are not sharp enough. Several newer and more advanced techniques have been proposed to address these problems. One interesting class of methods are the graph based methods [11] which has yielded several impressive results [12,13].

PCNN is a biologically inspired type of neural network, which based on the experimental observations of synchronous pulse bursts in the cat and the monkey visual cortex [14]. Compared with previous classical Artificial Neural Networks model, PCNN can be applied to image processing without training process. However, it needs to properly set a number of numerical control parameters for which it is usually not known a priori how to select the best values for a given application. The optimum values typically depend on attributes of the images to be segmented, e.g. pixel intensity ranges, contrasts and noise levels. As a consequence, values successful for one set of images may not be useful for other images with different intensity ranges, contrasts and noise levels. A number of different solutions have been suggested to address this problem, including the use of adaptive connection weights between neighboring neurons [15], automatic adaptation by genetic algorithms [16], reinforcement learning [17] and neural networks [18].

This paper proposes an adaptive segmentation method based on a modified PCNN, which multi-thresholds determined by the water region area in a histogram. Those control parameters can be achieved adaptively. A number of injection images are segmented. According to continuity and smoothness properties of extracted objects' moving traces, the inspection machine judges whether this injection is acceptable. Furthermore, improved spin/stop technique of controlling motors and illumination styles are applied through a large quantity of experiments to reduce the influence of air bubbles. The experimental results show that the inspection machine is superior to proficient workers, and can detect visible foreign particles effectively with satisfactory speed and accuracy.

In the following paragraphs, Section 2 introduces the architecture of the PCNN model. Section 3 gives intelligent inspection machine's overview. Section 4 brings forward key algorithms of foreign substances detection. Section 5 is devoted to the experiments and analysis of their results. Section 6 gives the conclusion.

## 2. Architecture of the PCNN Model

PCNN is a biologically inspired type of neural network, which is based on the experimental observations of synchronous pulse bursts in the cat's visual cortex by Eckhorn *et al.* and was adapted for image processing by Johnson [19].

### 2.1. PCNN Neuron Model and Parameters Determination

**Figure 1.**
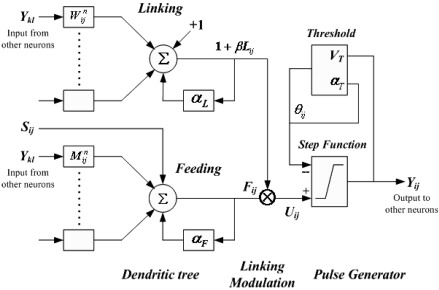
Structure of a PCNN neuron.

The PCNN is significantly different from other artificial neural network models in both its structure and operation. There is no training phase involved. Each neuron in the processing layer is directly tied to an input, in this case an image pixel or a set of neighboring image pixels. These are the feeding inputs, and they are also linked to nearby neurons, the linking inputs. The feeding inputs are iteratively processed together with the linking inputs producing a pulse train. The PCNN neuron consists of three parts [19,20]: the dendritic tree, the linking modulation, and the pulse generator, as shown in Figure 1.

The corresponding neuron's functions are:

(1) $$F_{\text{ij}}[n]=e^{-\alpha_{F}\delta_{n}}\cdot F_{\text{ij}}[n-1]+S_{\text{ij}}+V_{F}\sum_{\text{kl}}M_{\text{ijkl}}Y_{\text{kl}}[n-1]$$

(2) $$L_{\text{ij}}[n]=e^{-\alpha_{L}\delta_{n}}\cdot L_{\text{ij}}[n-1]+V_{L}\sum_{\text{kl}}W_{\text{ijkl}}Y_{\text{kl}}[n-1]$$

(3) $$U_{\text{ij}}[n]=F_{\text{ij}}[n]\cdot (1+\beta L_{\text{ij}}[n])$$

(4) $$Y_{\text{ij}}^{}[n]=\{\begin{array}{ccc} 1 & \text{if} & U_{\text{ij}}[n]>\theta_{\text{ij}}[n-1]\\ 0 & & \text{otherwise} \end{array}$$

(5) $$\theta_{\text{ij}}[n]=e^{-\alpha_{\theta }\delta_{n}}\cdot \theta_{\text{ij}}[n-1]+V_{\theta }\cdot Y_{\text{ij}}[n]$$

where, ***F****_ij_* - feeding input; ***L****_ij_* - linking input; ***U****_ij_* - internal state; ***S****_ij_* - external stimulus; ***θ****_ij_* - dynamic threshold; ***M*, *W*** - synaptic weights matrix to neuron; ***V****_F_*, ***V****_L_* and ***V****_θ_* - normalizing constant; ***α****_F_*, ***α****_L_* and ***α****_θ_* - negative decay constants of leaky integrator; ***β*** - linking strength between neurons; ***n*** - iteration times; ***Y****_ij_* - output.

A PCNN is a two-dimensional non-training neural network in which each neuron in the network corresponds to one pixel in an input image. The neuron receives its input (e.g. intensity) as an external stimulus. However, each neuron also connects with its neighboring neurons, receiving local stimuli from them. These stimuli are combined in an internal activation system, and are accumulated until they exceed a dynamic threshold. This will result in a pulse output, which is called natural fire. Through an iterative process, the algorithm produces a temporal series of binary images as outputs. Due to the linking of neighboring neurons, segments of the image consisting of pixels of similar intensity values tend to pulse together, which is called captured fire. The output of a PCNN can therefore be used for image segmentation by taking the pixels corresponding to synchronously pulsing neurons as segments.

Nevertheless, the performance of image segmentation based on PCNN depends on the suitable PCNN parameters. It is necessary to adjust the various threshold parameters of its mathematical model manually and then can achieve the optimum processing. As Karvonen [21] mentioned, a very large set of data should be required to optimize PCNN parameters, which is unfeasible in most applications. Hence, in order to determine PCNN parameters adaptively, this paper suggests an adaptive segmentation method based on a modified PCNN, which multi-thresholds determined by water region area in histogram. The implementation of the algorithm is computationally simple and can be used in foreign particles real-time detection in injections. The detailed process is given as following.

### 2.2. Multi-Threshold Acquisition Using “Water Region Area” Method

A novel threshold auto-detection algorithm in image histograms is proposed. In accordance with the intuitional features of the histogram, the peaks of the histogram are considered as watersheds, each valley including two neighboring peaks and a valley bottom points. We call the maximal water capacity in each valley the “water region area”.

Step 1: Draw image histogram and normalize them.
Step 2: Seek all peaks and valley bottom points in the histogram.
Step 3: Calculate the water region area from the left valley bottom point. Define ***θ*** that lies within [0.01, 0.05]. The smaller ***θ*** is, the more threshold points we will get. When the water region area is larger than ***θ***, the corresponding valley bottom point will be kept in threshold array ***T****_m_*. Meanwhile, the corresponding left side peak point will be kept in peak points array ***P****_m_*. Otherwise the valley will be taken as invalid. At this situation, comparing the two peaks' values located in the valley's two sides:
  1. If the left peak point is larger than the right one, it will be treated as the new left peak point. While the next right peak point will be the new right peak one, the smaller between the current and the next valley bottom point will be regarded as the new valley bottom point.
  2. Otherwise, the right peak point, the right valley bottom point and the next right peak point will be regarded as new left peak point, new valley bottom point and new right peak point respectively and then the new water region area will be calculated again.
Step 4: Iteratively execute Step3 until all valley bottom points have been processed and then we can get the threshold array ***T****_m_* (***m*** = 1, …, ***M*** and ***T****_1_*<…< ***T****_m_*) and the corresponding peak array ***P****_m_* (***m*** = 1, …, ***M*+*1*** and ***P****_1_* <…< ***P****_M_***_+_***_1_*). Hence, a valid valley ***V****_m_* includes two neighboring peaks {***P****_m_*, ***P****_m_***_+_***_1_*} and a threshold Tm (***P****_m_* < ***T****_m_* < ***P****_m_***_+_***_1_*). Figure 12 in Section 5 shows water regions and corresponding thresholds of images with black and white foreign particles respectively.

### 2.3. Modified PCNN Model

**Figure 2.**
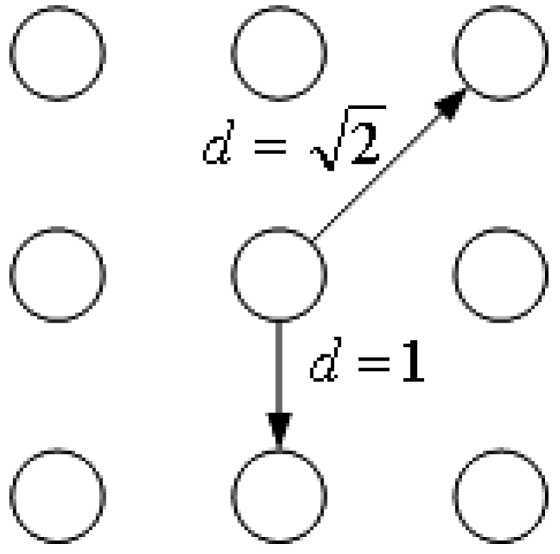
Linking distance between two neurons.

Considering the applications of foreign particles segmentation in injections, the PCNN model we have applied is a modification of the original PCNN [19], adapted slightly from Karvonen's model [21], and is implemented by applying iteratively the equations:

(6) $$L_{\text{ij}}[n]=\sum w_{\text{ijkl}}Y_{\text{kl}}[n-1]$$

(7) $$U_{\text{ij}}[n]=S_{\text{ij}}(1+\beta L_{\text{ij}}[n])$$

(8) $$Y_{\text{ij}}[n]=\{\begin{array}{ccc} 1, & & U_{\text{ij}}[n]>T_{\text{ij}}[n]\\ 0, & & \text{otherwise} \end{array}$$

where ***T****_ij_* is a threshold value. They are a set of fixed threshold values, ***T****_m_* (m = 1, …, ***M***) determined by water region area method mentioned above. Linking weight ***w****_ijkl_* is determined by Function (9), which satisfies human vision system (HVS) the best after many experiments' comparisons:

(9) $$w_{\text{ijkl}}=\{\begin{array}{ccc} 1 & \text{if} & 0\leq d_{\text{ijkl}}\leq 1\\ e^{1-d_{\text{ijkl}}} & \text{if} & d_{\text{ijkl}}>1 \end{array}$$

where, ***d****_ijkl_* is the linking distance between neuron (***i, j***) and (***k, l***), as shown in Figure 2.

Starting with the biggest threshold ***T****_M_*, an object whose mean gray value is larger than ***T****_M_* will be picked out at the first iteration. We keep the threshold ***T****_M_* fixed during the following iterations until no firing happens. After the first iteration loop, both the natural firing pixels and capture firing pixels are collected, which is the first level PCNN segmented objects with the largest gray value. Then the second level objects can be got by the same algorithm using threshold ***T****_M-1_*. Repeating this progress until all thresholds are processed.

Considering those pixels whose intensities are smaller than peak point ***P****_m_*, ought not to be captured at ***T****_m_* even if they have the largest linking value 1, so in the iteration loop at ***T****_m_*, the value of ***β****_m_* is:

(10) $$\beta_{m}=\frac{T_{m}}{P_{m}}-1$$

Because the least peak point ***P****_m_* may be 0, we choose the corresponding ***β****_m_* to be 0.1- 0.3 at this situation.

## 3. Intelligent Inspection Machine Overview

### 3.1. Types of Foreign Substances

According to the source, foreign particles in injection liquids can be classified into fibers, glass particles, rubber, hair and styrene resin, etc; but depending on their appearance particles can be divided into only two types, black and white.

Sometimes the diameters of these foreign substances are about 0.05 mm. This is too small to be easily seen by human eyes. However, they can be detected under special illumination. Some appear as black spots against diffused illumination (silhouette illumination). Others appear as luminous spots when illuminated by collimated light. Table 1 shows the typical samples and sources of the foreign particles.

### 3.2. System's Mechanical Structure

The intelligent inspection machine acquires every online injection's image sequence with a CCD camera, applying effective algorithms to detect foreign particles and giving a rejection signal to a main control system. Hence, a functional image acquisition part, including mechanical and electrical units, plays a key role in the system.

**Figure 3.**
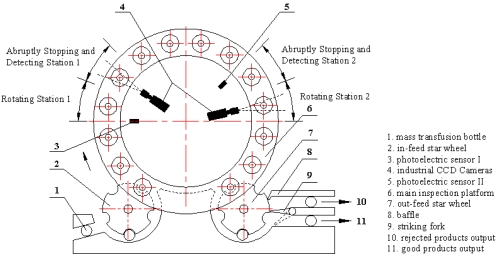
Top view of inspection system's rotary table architecture.

**Figure 4.**
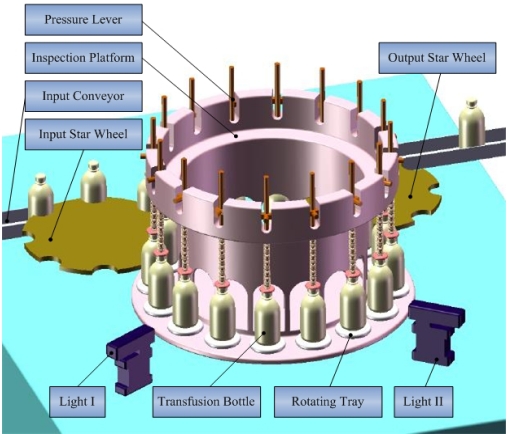
Three-dimensional drawing of the Automatic Inspection Machine.

Distinguishing between two completely different kind of foreign particles with just one illumination style can only increase the detection algorithm's complexity. Therefore, two detecting stations are arranged as shown in Figure 3 and Figure 4.

A rotary table mechanism is applied in system's mechanical design. An in-feed star wheel, main inspection platform and out-feed star wheel constitute the whole transmission system. Two important stations are defined as follows:

1. High speed revolving station. Rotating tray, driven by the motor which is located underneath the machine table, rotates quickly as the injection bottle triggers the photoelectric sensor 1. The foreign particles can be moved to the center of the bottle from the liquid surface, the valley bottom and the side walls of the bottle. To reduce the occurrence probability of the air bubbles deriving from bottle's violent vibration, designed rotating tray and pressure lever guarantee its steady rotation and high quality image sequence can be acquired. Detecting station 2 is similar.
2. Abrupt stopping station. One of areas arranged for detection needs. Bottles stop abruptly upon entering this area. Due to inertia, only the solution moves and forms a vortex for a while in the bottle while cameras acquire an image sequence with their external capture mode for the foreign particle detection that follows.

### 3.3. Electric Control System

**Figure 5.**
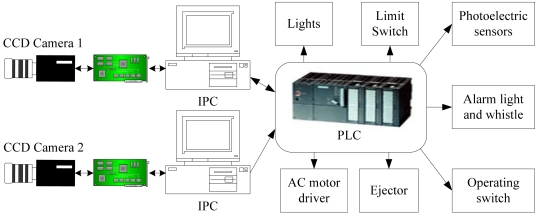
Electric configuration of intelligent inspection machine.

Figure 5 shows the electrical configuration of the intelligent inspection machine. A control system with industrial PC and PLC has better real-time performance and stability, which fits the assembly's long time working situation completely. IPC is mainly in charge of running the detection software, image analysis and product qualification's judgment. Especially, a PLC sends trigger pulses to the camera to every 200 milliseconds as the relevant photoelectric sensor is trigged by a passing injection bottle. IPC accesses the images stored in video capturing card via PCI bus, judging the injection qualified or not with the detection software then sending signals to the ejector.

### 3.4. Illumination Styles

**Figure 6.**
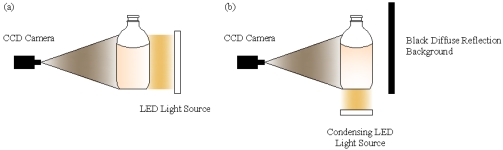
Illumination Styles: (a) Silhouette illumination. (b) Bottom light reflection illumination.

Illumination is the key part in the inspection machine. Good illumination can improve the system's resolution and simplify the detection algorithms; otherwise thorny problems like random spots, overexposure etc. can be caused. To acquire high-definition, stable luminance images, LED lights are selected. For detection of black foreign particles like hair, carbide, a 2-D arrayed LED panel shown in Figure 6(a) is put behind of the bottle facing the camera. High-contrast images can be achieved because of the particles' obstruction of light. To detect white particles like glass fragments, a round condensing LED is installed underneath the bottle and a plastic blackboard standing behind, as shown in Figure 6(b). Reflected lights can reach the camera as the glass fragment falls. Sample images can be found in Figures 10 and Figure 11.

### 3.5. Improved Spin/Stop Servo Control

**Figure 7.**
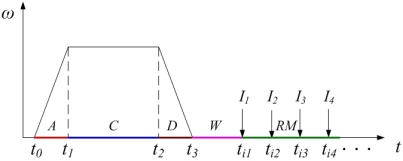
Improved spin/stop technique of the servo system.

To reduce the occurrence probability of air bubbles, an improved spin/stop technique of the rotating tray is demonstrated with reference to Figure 7, in which the abscissa represents the time (***t***), and the ordinate represents the spin speed (***w***).

The spin/stop technique is preferably executed as follows:

1. At ***t*** = ***t****_0_*, the bottle is accelerated with a substantially constant acceleration to a spin speed of 1000 revolutions per minute; this acceleration needs about 0.1 second. This phase is indicated as “acceleration phase ***A***”.
2. At ***t*** = ***t****_1_*, the acceleration is stopped, and the spin speed is maintained constant at 1,000 rpm for about 0.7 seconds to ***t****_2_*. This phase is indicated as “constant phase ***C***”.
3. Starting at ***t*** = ***t****_2_*, the bottle is brought to a stop with substantially constant deceleration in about 0.1 seconds. This phase is indicated as “deceleration phase ***D***”.
4. After the bottle has come to a stop (***t****_3_*), possible foreign particles are allowed some time ***t****_w_* to move to the center, while the air bubbles that possibly appeared are allowed some time to float upwards, before starting any measurements. This phase is indicated as “wait phase ***W***”. A suitable waiting time ***t****_w_* is about 0.2 seconds.

Experiments show that small foreign particles do not appear visibly at all times, so subsequent images should be taken and compared to improve the detection reliability. This is also illustrated in Figure 7, where the moments in time where images ***I****_1_*, ***I****_2_*, ***I****_3_*, etc. are provided are indicated as ***t****_i1_*, ***t****_i2_*, ***t****_i3_*, etc. respectively. This phase is indicated as “repeated measurement phase ***RM***”. Hence, chances that a foreign particle is invisible to the camera in all images are decreased substantially due to the repeated detection cycles.

## 4. Key Algorithms of Foreign Substances Detection

**Figure 8.**
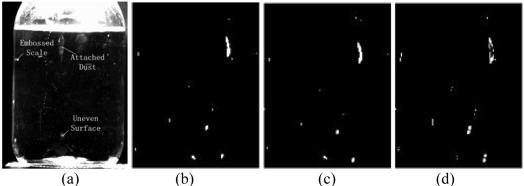
(a) Glucose injection's surface defects. (b), (c) are captured images (d) absolute difference between (b) and (c)

**Figure 9.**
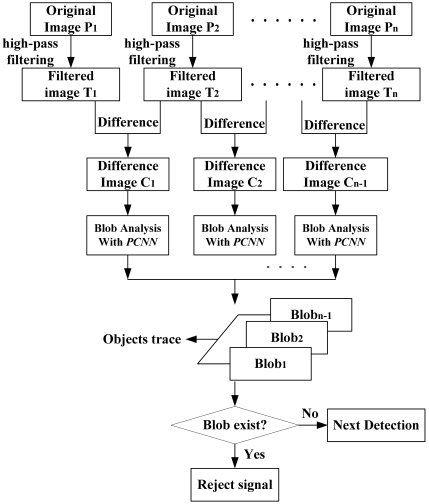
Foreign substances detection flowchart.

The majority of injection bottles used in China do not strictly meet quality standards. Fingerprints, graduations, dust, can sometimes be seen on the surface of the bottles. Even some foreign substances or bubbles, shown in Figure 8(a), can be found in the glass walls, which cause lots of difficulties for detection. In a single image, the foreign particles lack structural information and distinct characteristics, especially they are small and usually occupy only several or dozens of pixels in the image. It is hard to distinguish them from noise (lights) and disturbances (dust or graduations) just depending on their gray values. Hence, special foreign particle detection algorithms should be used. Foreign substances in liquid have two obvious properties: the moving trace is continuous over time and there are certain gray value differences between foreign particles and background, so a sequence image difference algorithm based on spatiotemporal continuity is proposed to remove existing static disturbances in the background, extracting objects with modified PCNN and performing the judgment according to the trace. A detection flowchart is shown in Figure 9.

### 4.1. Image Preprocessing

In the image with foreign objects, the majority of the pixels are occupied by background which belongs to slowly varying low frequency parts, while the foreign particles are high frequency parts and they are unrelated with the background. Hence, low frequency components can be inhibited with a high pass filter letting high frequency ones pass, nevertheless some parts of the high luminance noise is kept.

Image M*N can be recognized as formed with large low frequency background and some high frequency parts. Suppose the captured sequence images are:

(11) $$f(x,y;t_{i})=p(x,y;t_{i})+q(x,y;t_{i})x=0,1,\ldots ,M;y=0,1,\ldots ,N;i=0,1,\ldots ,n$$

where, ***p*(*x, y; t****_i_***)** are ideal background; ***q*(*x, y; t****_i_***)** are image details including high frequency noise and foreign particles. According to convolution theorem:

(12) $$G(u,v;t_{i})=F(u,v;t_{i})*H(u,v;t_{i})$$

where ***F*(*u, v; t****_i_***)** is the Fourier transformation of original images; ***H*(*u, v; t****_i_***)** is the transfer function; ***G*(*u, v; t****_i_*) is the high-pass filtering output images after Fourier transformation, then reverse transform it and get the enhanced images. Butterworth high-pass filter is applied in this article.

### 4.2. Sequential Images' Difference

Assuming ***P****_1_****, P****_2_****, …, P****_n_* are ***n***-frame original images, ***T****_1_****, T****_2_****, …, T****_n_* are filtered sequence, latter frame subtract the former one and get ***n-1***-frame sequence ***C****_1_****, C****_2_****, …, C****_n-1_*. Only noise and foreign objects remain in the difference images:

(13) $$C_{k}=T_{l}-T_{l-1}\;k=1,2,\ldots ,n-1;l=2,3,\ldots ,n$$

To avoid the trivial possibility of a tiny spatial location difference moving objects on two frames, the camera's external asynchronous capture mode is employed. A PLC sends trigger pulses every 200 milliseconds. Figure 8(d) shows the absolute difference between two successive frames.

As we can see from the difference image, small particles do exist in the glucose injection liquids. However, injection liquids are chemical liquids which also contain some tiny medicinal powder. Referring to [1], elaborate requirements and demonstrations are defined on small objects' dimensions whether they can be accepted or not. To decide whether the bright spots, shown in Figure 8(d), are foreign substances, noise or acceptable tiny medicinal powders, more accurate object outlines should be obtained.

### 4.3. Foreign Substance Segmentation Based on Modified PCNN

Difference images are segmented with modified PCNN directly because of its superiority, omitting energy accumulation method described in [22], which improves the detection speed effectively.

### 4.4. Feature Extraction and Foreign Particles Judgment

To recognize multiple objects (shown in Figure 11), every particle in the sequence images should be labeled and their invariance properties must be extracted. Selected properties need perfect invariance to image geometrical transformations such as motion, rotations and translations. Ideal matching results can be achieved with proper values which have invariance properties. Three values, which are calculated in the Euclidean space, concluded from many experiments have good invariance property shown as follows:

1. Ratio of objects' circumferences to areas, **λ_1_. λ_1_** = **L_i_/S_i_**, L_i_ is circumference and **S_i_** is the area of the particle.
2. Length-width ratio **λ_2_**. Searching each object's minimum circumscribed rectangle **R_i_**, which length is a_i_, width is b_i_. **λ_2_** = **a_i_/b_i_**.
3. Compactness **λ_3_. λ_3_** = S_i_ /(a_i_*b_i_).

Furthermore, coordinates (**C_ix_, C_iy_**) of particles and remaining noise' centroids are calculated. ***f*(x, y)** is the gray value:

(14) $$C_{\text{ix}}=\frac{\sum_{x,y\in R_{i}}xf(x,y)}{\sum_{x,y\in R_{i}}f(x,y)},\;C_{\text{iy}}=\frac{\sum_{x,y\in R_{i}}yf(x,y)}{\sum_{x,y\in R_{i}}f(x,y)}$$

Because of **λ_1_, λ_2_, λ_3_**'s good invariance properties, there are almost no changes in the sequence images. Hence, points which have shortest distance between images' proper values are the best matched ones. Distance can be calculated with Formula (15). We note the corresponding point's coordinates and connect them into a line:

(15) $$d=\sqrt{{\left(\lambda_{1}^{i+1}-\lambda_{1}^{i}\right)}^{2}+{\left(\lambda_{2}^{i+1}-\lambda_{2}^{i}\right)}^{2}+{\left(\lambda_{3}^{i+1}-\lambda_{3}^{i}\right)}^{2}}$$

According to object's moving trace, we can judge whether it is foreign substance or caused by noise through two principles:

1. Images are captured when the bottle are kept static. At this moment, foreign particles are falling and their centroids' ordinates become larger (suppose the origin of coordinates is located in the top left corner).
2. Traces formed by particles are smooth, while which generated by noise are unordered.

So, one smooth trace with objects' ordinates increasing indicates the existence of the foreign particles.

## 5. Experiments and Analysis

To evaluate designed intelligent inspection machine and detection software's effectiveness, the “Knapp-Kushner” testing programs which are recognized by European pharmacopoeia and U.S. Food & Drug Administration (FDA), are applied. Testing results are shown in Table 2. In the following experiments, 0.9% sodium chloride solution and 10% glucose are selected as the testing objects. Hardware: CPU (Pentium 4, 3.00 GHz), 1 GB memory, 659×494 full frame resolution digital progressive scanning CCD camera UP-680, 16 mm Computar lens, condensing LED SPL-8-5-R, back light source FL-100-20-R.

### 5.1. Experiment 1 (Black Foreign Substance Detection)

**Figure 10.**
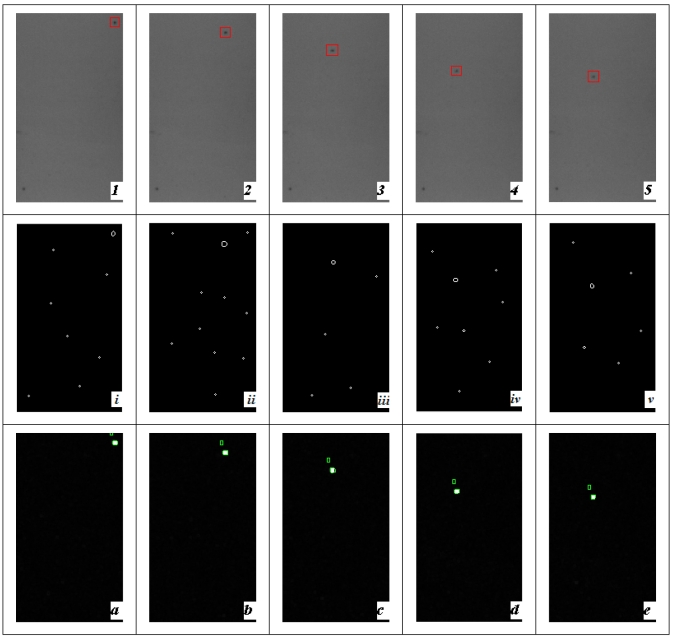
Black foreign particle segmentation with back illumination (1-5) sequential images every 200 ms (the carbide is labeled with red rectangle). (i-v) Segmentation results with Canny operator. (a-e) Segmentation results with modified PCNN (falling black objects are labeled)

Five sequential images of 0.9 % sodium chloride injection were captured with back illumination and doing the segmentation with the Canny operator and modified PCNN, respectively, which is shown in Figure 10. Here, an edge-based method (“Canny operator”) was selected as an example to separate two adjacent regions with detected edge lines, comparing with proposed modified PCNN method. Their shortcomings can be concluded from the comparisons.

### 5.2. Experiment 2 (White Foreign Substance Detection)

**Figure 11.**
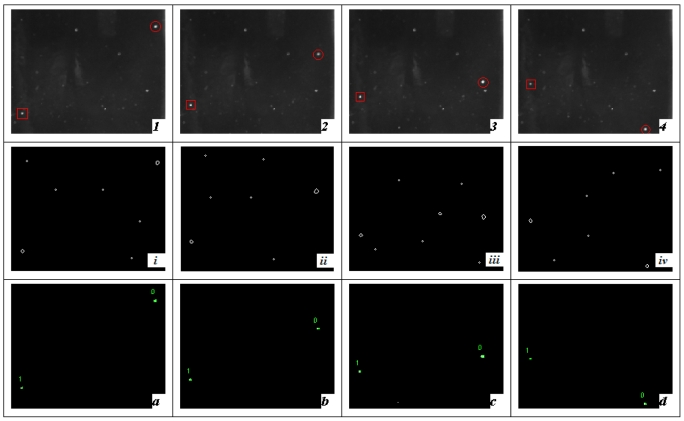
White foreign substances (glass chip) segmentation with bottom light (1-4) sequential images every 200 ms (bubble and glass chip are labeled with red rectangle and circle respectively). (i-iv) Segmentation results with Canny operator. (a-d) Segmentation results with modified PCNN (falling glass chip and rising bubbles are labeled).

**Figure 12.**
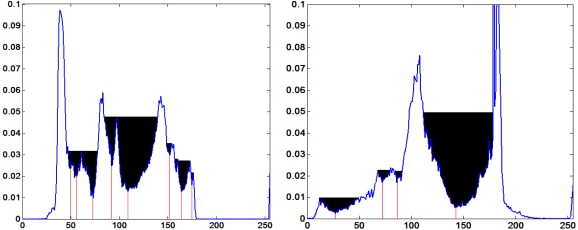
Water regions and thresholds of first row images in Figures 10 and 11, respectively.

Capturing four sequential images of 0.9% sodium chloride injection with bottom illumination and doing the segmentation with Canny operator and modified PCNN respectively, which is shown in Figure 11.

### 5.3. Experiment 3 (Algorithm Running Time)

### 5.4. Experiment 4 (Injections with Typical Foreign Particles Detection Test)

Proficient workers select 112 bottles of 10% glucose injection with typical foreign particles which are then checked by the designed inspection machine. Its correct detection rate is about 99.1% which can be considered effective. Checking results is shown in Table 4; here the parameters “sensitivity” denotes inspection accuracy, “qualified” and “unqualified” denote numbers of judgment by the inspection machine.

### 5.5. Experiment 5 (Batch Detection with Machine)

This test is arranged for 4 days and divided into 2 steps, 10% glucose injections as batch detection objects. (i) A batch of glucose injections are inspected by machine and then the qualified/unqualified products will be re-inspected by proficient workers on four different days. The result is shown in Table 5. (ii) First, injections are inspected by proficient workers, then the qualified products will be re-inspected by machine to test the human inspectors' omission error rate over four different days. The rejected ones will be reconfirmed by workers. Result is shown in Table 6.

### 5.6. Experiments Analysis

As shown in Experiments 1-3, foreign particles' edges can be extracted with the Canny operator, nevertheless high-frequency random noise detected too, which disturbs the judgment whether it could be accepted or not. Sometimes noise is assumed to be foreign particles and a sample rejected, improving the error detection rate. While the modified PCNN model filtered noise effectively, judging the extracted blobs as foreign substances according to blobs' moving continuity and trace consistency. Based on running time, the modified PCNN is superior to traditional ones; commonly 5-8 fold iteration can reach reasonable segmentation results. Usually the running time is about 0.05 s.

Table 2 and Experiments 4 and 5 indicate that the intelligent inspection machine's detection effects for injection are superior to the traditional inspection by human eyes. Omission error rates are only one fourth those of the proficient workers. Mechanical running property is steady with low bottle crushing rates.

## 6. Conclusions

This article describes a real-time visual based automatic intelligent inspection system for foreign particles in injection liquids. Obstructions to detection due to unevenness such as scratches, embossed symbols, and graduations on the bottle surface are removed with sequence image processing. Parameters of PCNN model are achieved adaptively by water region area in histogram and the segmentation results are effective. It is capable of perfectly segmenting images even when there is a considerable overlap in the intensity ranges of adjacent regions. The inspection machine judges the injection qualified or not according to the continuity and smoothness properties of extracted objects' moving traces.

The correct detection rate is at least about 99.1%, which was confirmed through lots of experiments. Other tests show that intelligent inspection machine's detection effects for injection are superior to those of proficient workers.

Future work may focus on pharmaceutical management guided by the inspection machine and design higher precision servo driving system to reduce the influence brought by the vibration deriving from the mechanical system. More suitable illumination styles and different image processing algorithms should be explored with the current inspection platform.

## Figures and Tables

**Table 1.** Foreign substance classification.

**Type**	Rubber	Glass particles	Fiber	Color spot	Hair
**Color**	White	White	White	Random	Black
**Source**	Bottle packing	Bottle collision	Cleaning	Bottle packing	workers
**Sample**	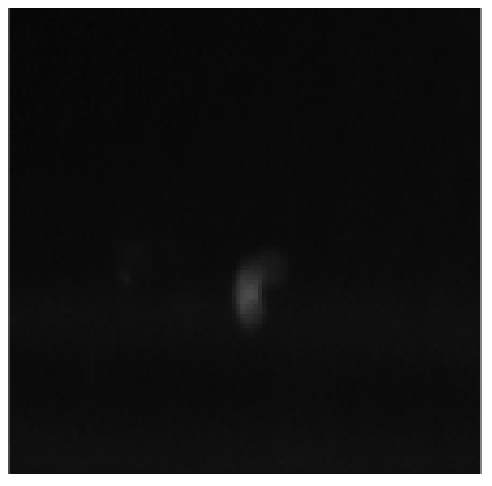	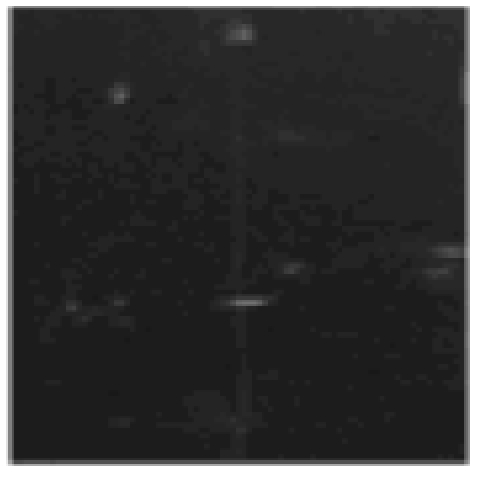	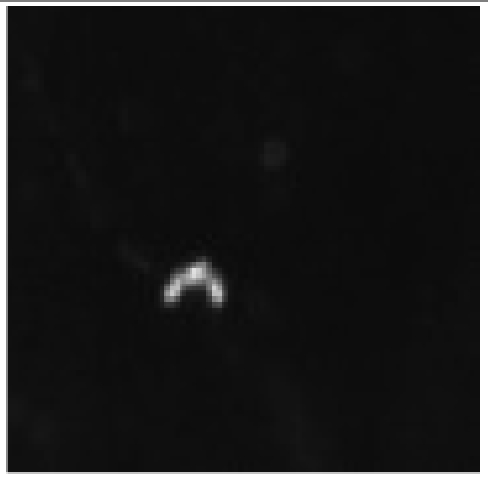	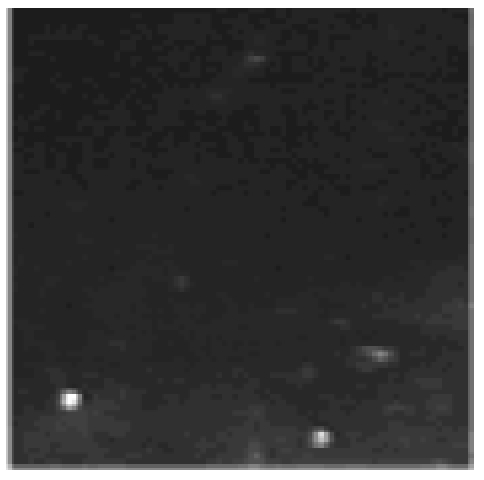	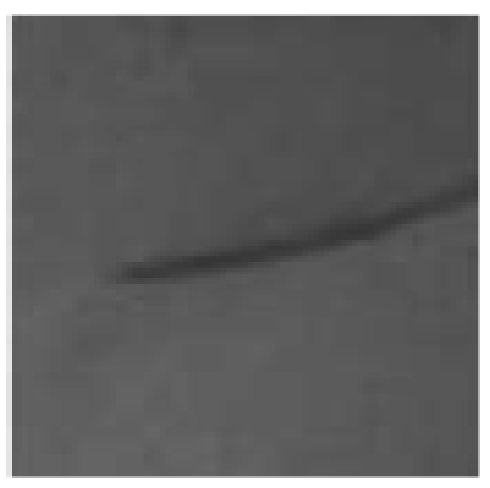

**Table 2.** Knapp-Kushner testing results.

**Sensitivity**	**FQA**	**FQB**	**FQB/FQA**	**Judging criterion**	**Result**
35	107	545	5.093	FQB/FQA > 1	Machine detection is superior to humans

Phrases definition in Table 2:

***Sensitivity***: one of parameters of the inspection machine.

***FQ****_i_*: ***i*** th bottle's factor of quality.

***FQ****_i_* = (n/N)*10, where ***n*** is rejected times; ***N*** is the overall detection time.

***FQA: FQ*** of proficient workers.

*FQA*_[7,10]_ = ∑ *FQAi*, only ***FQ****_i_* belongs to [7, 10] are added.

***FQB: FQ*** of inspection machine.

*FQB*_[7,10]_ = ∑ *FQBi*, only ***FQ****_i_* belongs to [7, 10] are added.

**Table 3.** PCNN running time (s).

**Image**	*n* = **4**		*n* = **32**		*n* = **256**	
	**PCNN**	**Modified PCNN**	**PCNN**	**Modified PCNN**	**PCNN**	**Modified PCNN**
With black particles	0.208	0.015	0.987	0.084	7.622	0.556
With glass chips	0.284	0.044	1.611	0.136	12.175	0.982

**Table 4.** Checking results with inspection machine.

**Checking times**	**Sensitivity**	**Qualified**	**Rejected**	**Correct detection Rate (%)**
1	5	1	111	99.1
2	5	0	112	
3	5	1	110	

**Table 5.** Workers' re-inspection result.

Sensitivity	*A*	*B*	*C*	Crushed number	*D* (%)	*E* (%)
12	53 550	44 130	54	7	0.12	0.013
12	50 990	43 132	68	5	0.16	0.010
12	51 056	41 075	48	6	0.12	0.012
12	50 976	40 118	55	8	0.14	0.016

Here, ***A***: overall number of injections to be detected; ***B***: qualified number judging by inspection machine; ***C***: rejected number out of ***B*** according to workers' re-inspection; ***D***: omission error rates of machine; ***E***: crushing rates.

**Table 6.** Re-inspection results of machine using qualified products by workers.

*X*	*Y*	*Z* (%)
14 980	69	0.46
15 240	71	0.47
14 928	77	0.52
15 120	73	0.48

Here, ***X***: overall number of injections inspected by proficient workers; ***Y***: rejected number judging by inspection machine and re-confirmed by workers; ***Z***: omission error rates of workers.
